# Automatic Colorectal Cancer Screening Using Deep Learning in Spatial Light Interference Microscopy Data

**DOI:** 10.3390/cells11040716

**Published:** 2022-02-17

**Authors:** Jingfang K. Zhang, Michael Fanous, Nahil Sobh, Andre Kajdacsy-Balla, Gabriel Popescu

**Affiliations:** 1 Quantitative Light Imaging Laboratory, University of Illinois at Urbana-Champaign, Urbana, IL 61801, USA; jzhng170@illinois.edu (J.K.Z.); mfanous2@illinois.edu (M.F.); 2 Beckman Institute for Advanced Science and Technology, University of Illinois at Urbana-Champaign, Urbana, IL 61801, USA; 3 Informatics Programs, University of Illinois at Urbana-Champaign, Urbana, IL 61801, USA; 4 Department of Bioengineering, University of Illinois at Urbana-Champaign, Urbana, IL 61801, USA; 5 Center for Artificial Intelligence Innovation, National Center for Supercomputing Applications, Urbana, IL 61801, USA; sobh@illinois.edu; 6 Department of Pathology, University of Illinois at Chicago, Chicago, IL 60612, USA; aballa@uic.edu; 7 Department of Electrical and Computer Engineering, University of Illinois at Urbana-Champaign, Urbana, IL 61801, USA

**Keywords:** spatial light interference microscopy, label-free, mask R-CNN, deep learning, automated colorectal cancer screening

## Abstract

The surgical pathology workflow currently adopted by clinics uses staining to reveal tissue architecture within thin sections. A trained pathologist then conducts a visual examination of these slices and, since the investigation is based on an empirical assessment, a certain amount of subjectivity is unavoidable. Furthermore, the reliance on external contrast agents such as hematoxylin and eosin (H&E), albeit being well-established methods, makes it difficult to standardize color balance, staining strength, and imaging conditions, hindering automated computational analysis. In response to these challenges, we applied spatial light interference microscopy (SLIM), a label-free method that generates contrast based on intrinsic tissue refractive index signatures. Thus, we reduce human bias and make imaging data comparable across instruments and clinics. We applied a mask R-CNN deep learning algorithm to the SLIM data to achieve an automated colorectal cancer screening procedure, i.e., classifying normal vs. cancerous specimens. Our results, obtained on a tissue microarray consisting of specimens from 132 patients, resulted in 91% accuracy for gland detection, 99.71% accuracy in gland-level classification, and 97% accuracy in core-level classification. A SLIM tissue scanner accompanied by an application-specific deep learning algorithm may become a valuable clinical tool, enabling faster and more accurate assessments by pathologists.

## 1. Introduction

From benign adenomatous polyps to carcinoma, colorectal cancer develops through a series of genetic mutations over a 5–10 year course [1]. The mortality of colorectal cancer is significantly reduced if this disease can be diagnosed at an early “localized” stage. The 5-year survival rate for patients with their disease diagnosed at the localized stage is 89.8%, while the same rate for patients with distant metastasis (late-stage cancer) drops to 12.9% [2]. In the United States, the preferred form of screening is a colonoscopy. In the 50–75 age group, 65% of individuals received colorectal cancer screenings in 2010, compared to 54% in 2002 [3]. As colorectal cancer has become the third most prevalent cancer diagnosed in the U.S., the American Cancer Society estimates that there will be 149,500 new cases in 2021 and 52,980 associated deaths [4]. The U.S. Preventive Services Task Force (USPSTF) recommended in 2016 [5] that all 50–75 year old adults receive colorectal cancer screening, but then in 2020, it issued a revised draft, which notably decreased the starting age for all adults’ screenings to 45–49 [6]. Among all the individuals receiving colonoscopies, the prevalence of adenoma is 25–27%, and the prevalence of high-grade dysplasia and colorectal cancer is just 1–3.3% [7,8]. However, 50% of all colonoscopy patients underwent a polyp removal or biopsy, as current screening tools cannot effectively distinguish adenoma from a benign polyp [9]. Following the procedure, pathologists examine the excised polyps to identify whether the tissue is cancerous or benign. As more individuals are expected to receive colonoscopies in the future, pathologists will likely see a significantly heavier workload. For the purposes of large-scale efficient screening, it is imperative to acquire new technologies that can decrease manual work by performing automated tissue investigation. The Papanicolaou test (pap smear) for cervical cancer screenings is a successful precedent, which employs semi-automated computational tools, and in the example of FDA-approved BD Focal Point Slide Profiler™, decreases pathology caseloads by 25% by detecting benign cases [10]. It must be noted, however, that the proper operation of such systems requires staining procedures tailored for narrow calibration thresholds, and continuous quality reviews are required throughout the machine’s operation [11].

Quantitative phase imaging (QPI) [12] has been established as a valuable label-free method for various biomedical imaging applications. Due its sensitivity to tissue nanoarchitecture and the ability to provide quantitative information from 3D and anisotropic tissue structures [13,14,15], QPI has been applied to different pathology problems in recent years [11,12,16,17,18,19,20,21,22]. Spatial light interference microscopy (SLIM) has been used as the core technology for label-free whole slide imaging (WSI), which revealed that the tissue refractive index is an effective intrinsic marker for pathological diagnosis and prognosis [14,16,19,20,23,24,25,26]. It was shown that tissue scattering coefficients computed from QPI data were able to predict disease recurrence after prostatectomy surgery [19,20]. Furthermore, it has been demonstrated that SLIM could provide information on collagen organization, which represents valuable information for disease stratification in breast cancer pathology [16,17,24].

Although this approach of “feature engineering” has the advantage of generating physically insightful markers [27] (e.g., scattering anisotropy factors, refractive index variance), it encompasses a limited range of the parameters that can be retrieved from these data [28]. There is always the possibility that certain clinically valuable parameters remain unevaluated. In recent years, the biomedical community has applied artificial intelligence (AI) techniques to data processing, visualization, and analysis [23,28,29,30,31,32,33,34,35]. Contrary to feature engineering, deep learning convolutional neural networks extracts a large number (millions) of features, including edges, pixel intensities, variations in pixel values, etc., for each image. AI can recognize image patterns that might be too subtle for human eyes, and thus significantly improves clinical cancer screening and diagnosis. In this scientific area, we have recently demonstrated that the combination of SLIM and AI can screen colorectal tissue as cancerous vs. benign [28]. However, while very encouraging, this previous work required segmented individual glands as the input. In other words, a prerequisite for this procedure was the manual annotation of gland regions, which diminished the practical use of our method.

In order to advance our screening method and provide completely automatic classification, here we used a SLIM-based whole slide imaging tissue scanner together with a mask R-CNN deep learning network, to both segment the glands and classify cancer and benign tissues. This new method of automatic colorectal cancer screening uses intrinsic tissue markers, and thus is now of potential clinical value. Importantly, this method does not require staining or calibration. Unlike existing staining markers, the signatures that are derived from phase information can be shared across different instruments and laboratories, without modification. Furthermore, we eliminated the need for manual segmentation as a pre-requisite of the procedure.

## 2. Results and Methods

### 2.1. Label-Free Tissue Scanner

**Figure 1:**
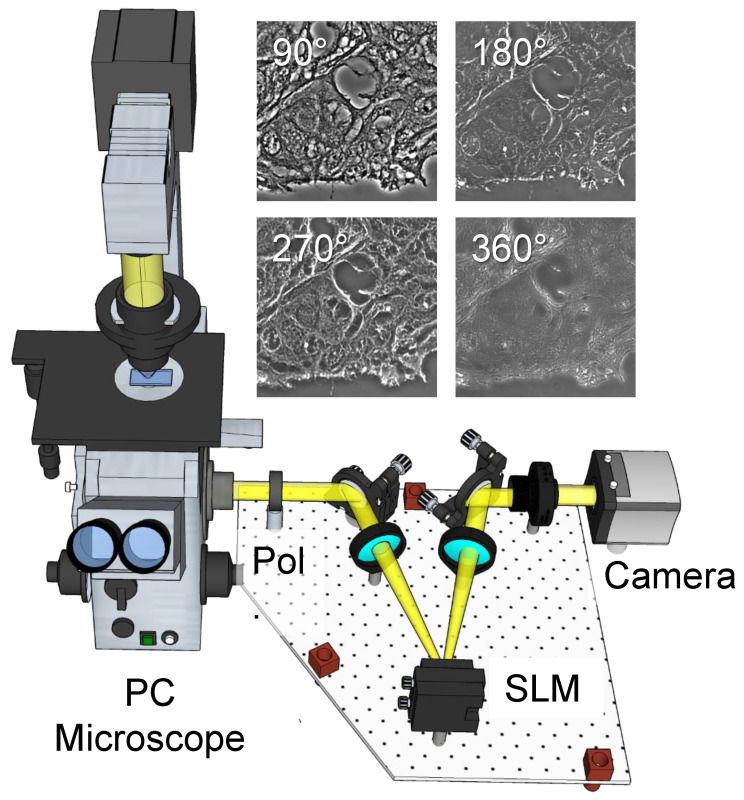
The SLIM tissue scanner setup. The SLIM system was implemented as add-on to an existing phase contrast microscope. Pol, polarizer; SLM, spatial light modulator. The four independent frames corresponding to the four phase shifts imparted by the SLM are shown for a tissue sample.

Our label-free WSI system consisted of a SLIM scanner with specially designed hardware and software [11]. As shown in Figure 1, the SLIM module (Cell Vista SLIM Pro, Phi Optics, Inc., Champaign, IL, USA) was an upgrade module to an existing phase-contrast microscope [36,37], which generates quantitative phase maps associated with the sample. Fundamentally, SLIM functions by making the ring in the phase-contrast objective pupil “tunable”. To achieve this, the image generated by a phase-contrast microscope was Fourier transformed at a spatial light modulator (SLM) plane, where the image of the phase-contrast objective ring was overlaid with the SLM phase mask, and the phase steps are shifted incrementally by 90° (Figure 1). From the four intensity images associated with the quarter wavelength phase shifts, the quantitative phase image was extracted to reveal the optical pathlength at each point in the field of view.

Equipped with novel acquisition software that pairs both CPU (central processing unit) and GPU (graphical processing unit) processing, the SLIM tissue scanner can acquire and process the four intensity images and display the phase image in real time [11]. The SLIM phase retrieval computation was performed at a different thread, while the microscope stage shifted the subsequent position. The scanning of a large field of view like a whole microscope slide and the assembling of the ensuing images into a single file were performed by our specifically designed software tools [11]. The final SLIM images were generated at maximum of 15 frames per second, as restricted by the refresh rate of the spatial light modulator. Our label-free scanner combines optical hardware and dedicated, highly parallelized software algorithms for data acquisition. TSLIM acquisition speed is in the range of existing commercial tissue scanners, which in turn only perform bright-field imaging. Throughout our experiments, a 40×/0.75 NA phase contrast objective was used for imaging. At this sampling rate (6.2 pixels/µm), the typical time for imaging a single tissue core (1 mm^2^ area) was approximately 12 sec. The industry standard is to compare 15 × 15 mm^2^ areas, which in our case required 1100 s for SLIM imaging [16]. On the other hand, a commercial instrument is ~4X faster, as it only provides bright-field. Furthermore, SLIM speed is orders of magnitude faster than other label-free modalities, such as IR and Raman.

### 2.2. Tissue Imaging

We used archival pathology material from 131 patients who went to the University of Illinois at Chicago (UIC) from 1993 to 1999 to receive colorectal resections for cancer treatment. Colon tissue cores 0.6 mm in diameter were collected from each patient, which corresponded to four groups: “tumor”, “normal mucosa”, “dysplastic mucosa”, and “hyper plastic mucosa” [11]. The tissue cores were finally transferred into high-density arrays for the purpose of imaging: primary colon cancer (127 cases), mucosa of normal colon (131 cases), dysplastic colon (33 cases), and hyperplastic colon (86 cases) [11].

From each specimen, two 4 μm thick sections were cut. The first section was deparaffinized and stained with hematoxylin and eosin (H&E) and imaged using the Nanozoomer (bright-field slide scanner, Hamamatsu Corporation, Bridgewater, NJ, USA). A pathologist made a diagnosis for all tissue cores in the TMA set, which was used as “ground truth” for our analysis. A second adjacent section was prepared in a similar way but without the staining step. These slides were imaged in our laboratory. All these studies complied with the protocols that the Institutional Review Board at the University of Illinois at Urbana-Champaign approved (see IRB Protocol no. 13900). Before imaging, the tissue slices were deparaffinized and cover-slipped in aqueous mounting media. A conventional microscope (Zeiss Axio Observer, 40×/0.75, AxioCam MRm 1.4 MP CCD) acquired a series of mosaic tiles of the tissues, which were then assembled into tissue microarray images. With ImageJ’s stitching functionality, we tiled high-resolution images of each core. Using Cell Vista SLIM Pro (Phi Optics, Inc., Champaign, IL, USA), 1.2 × 1.2 mm^2^ regions, consisting of 4 × 4 mosaic tiles, were SLIM-imaged at a 0.4 μm resolution, in four seconds. For each frame, stage motion takes 100 ms, SLM modulator stabilization 30 ms, and exposure 10 ms. From the resulting image file, 10,000 × 10,000 pixels images were cropped, corresponding to a field of view at 1587.3 × 1587.3 μm^2^.

For this work, only tumor and normal mucosa images were used, since there were not enough dysplastic mucosa (33 samples) and hyperplastic mucosa (86 samples) images to support our training. Between normal mucosa and cancer are hyperplasia and dysplasia. Hyperplasia is a normal response to external pressures and stimuli, and the morphology of the cells are still normal despite an increase in cells. Hyperplasia can be easily identified by a well-trained neural network. In dysplasia, the cells proliferate abnormally and appear disorganized. Dysplasia may likely revert to dysplasia or deteriorate into cancer. High-grade dysplasia increases the risk of developing colorectal cancer by up to 50%, as stated by The American Cancer Society. It indicates that dysplasia is common and exhibits very low malignant potential. Despite abnormality in the structure, dysplasia is still confined to its original epithelial layer. As a comparison, cancer is invasive, with the cancer cells invading neighboring tissues. A neural network can differentiate dysplasia from cancer, if fed with sufficient and diversified data, which will be the subject of future work.

From the 127 primary colon cancer images and the 131 normal colon mucosa images (Table 1), a total of 258 images were selected. Of them, 98 cancer images and 98 normal images were assigned for training, 15 cancer images and 15 normal images were assigned for validation, and 14 cancer images and 18 normal images were assigned for testing. As a result, 76% of the dataset was used for training, 12% for validation, and the remaining 12% for testing. The 32 testing images were hidden until all the training and fine-tuning was completed and finalized.

### 2.3. Deep Learning Network

We used deep learning for the semantic segmentation of tissue glands. For this purpose, three steps were required: (1) object recognition; (2) object localization; and (3) object classification [38]. During object detection, a bounding box with (x, y) coordinates, together with a class label were predicted for each object. During semantic segmentation, the input image was partitioned and every pixel value was predicted and assigned to a certain class label. In addition to semantic segmentation, instance segmentation added a pixel-wise mask to each object detected.

**Figure 2:**
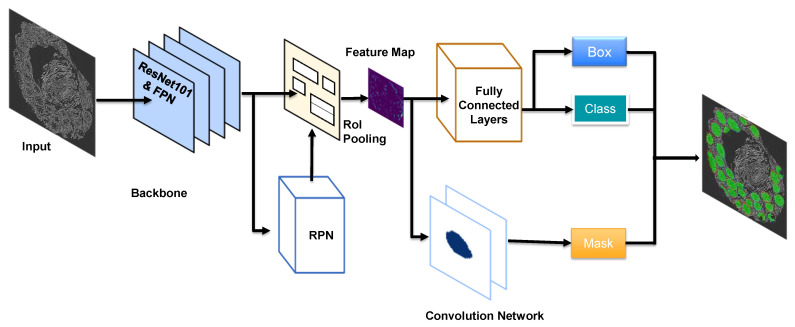
The mask R-CNN network architecture. The mask R-CNN (regional convolutional neural network) framework contains two stages: scanning images and generating regional proposals for possible objects; and classifying the proposals and generating bounding boxes and pixel-wise masks. This specific network adopts the backbone of ResNet101 plus FPN for feature extraction. The RPN (region proposal network) scans over backbone feature maps, which allows extracted features to be reused, and removes duplicate calculations. The RPN outputs two results for each anchor: anchor class (foreground or background: foreground implies the potential existence of an object) and bounding box refinement (the foreground anchor box, with its location and size, is refined to fit the object). The final proposals are passed to the next stage. At this stage, two outputs are generated for each ROI as proposed by the RPN: class (for objects) and bounding box refinement. An ROI pooling algorithm crops a piece of area from a feature map and resizes it to a fixed size, which enables the functionality of classifiers. From this stage, a parallel branch of two fully convoluted layers is added that generates masks for the positive regions that are selected by the ROI classifier. The other branch of fully connected layers takes the outputs of the ROI pooling and outputs two values: a class label and a bounding box prediction for each object.

A mask R-CNN deep learning network was set up with an algorithm that He et al. published in 2017, which is a further improvement on the previous R-CNN, fast R-CNN [39] and faster R-CNN algorithms [40]. In addition to the faster R-CNN architecture that predicts class labels and bounding boxes, mask R-CNN adds a two-CONV-layers branch (Figure 2) that predicts the mask for each detected object and gives a pixel-wise visualization of each object’s location. Our mask R-CNN network [41] uses Keras and TensorFlow and loads a pretrained ResNet101 network from the ImageNet database. The residual connections in this algorithm can improve gradient flow, and thus boost the training of deeper networks. With our dataset of 196 images for training and 30 images for validation, ResNet101, with its 101 layers, extracted valuable features and supported a high accuracy in gland detection and classification.

In our mask R-CNN network there were three classes: “cancer”, “normal”, and “background”, in which only “cancer” and “normal” were flagged. We trained the network using a single GPU (with 12 GB memory) and set the batch size to 1. The RPN (region proposal network) adopts (8, 16, 32, 64, 128) as anchor box scales and [0.5,1,2] as anchor ratios. 8, 16, 32, 64, and 128 are the length of square anchor sides in pixels, which means that 5 square anchor boxes of 8 × 8, 16 × 16, 32 × 32, 64 × 64, and 128 × 128 pixels were generated for each anchor point. With the three anchor ratios, a total of 5 × 3 anchor boxes were generated for each anchor point. We believed that such a combination of anchor scales and anchor ratios would ensure a satisfying performance in our purpose of gland detection. Anchor scales should be adjusted as per the overall shapes of the objects that are detected. Full-size masks were used throughout our training process, which meant that the masks were set to have the same width and height as the original image. Another alternative method is to use mini masks during training, which can be generated by extracting the bounding box of the object and resizing it to a pre-defined shape. For example, a full-size mask of 1024 × 1024 pixels can be resized to a size of 224 × 224 pixels, which reduces memory requirements during training. However, mini masks likely do not facilitate training performance in the case of small-size objects, such as cells, since minor rounding errors may arise while the masks are converted to a smaller size and then eventually converted back. The optimizer adopts ADAM, which is combined with an adaptive gradient algorithm (AdaGrad) and root mean square propagation (RMSProp). AdaGrad improves performance in cases with sparse gradients, while the RMSProp algorithm works well in cases with noise. The learning rate was 0.001, learning momentum was 0.9, and weight decay was 0.0001. A moderate level of image augmentation was implemented, which included horizontally flipping 50% of all images, vertically flipping 50% of all images, affine rotation by 90°, 180°, and 270°, and gaussian blur with 0.0–5.0 sigma.

The machine in this work had one GPU, NVIDIA RTX 2070. It took 46 h 30 min to complete 400 epochs of training. The first 50 epochs only trained top layers and the remaining 350 epochs were transferred to train all 101 layers. The images were annotated with the VGG Image Annotation tool, and data were written into a JSON file. The gland detection confidence probability was tested separately at 0.70, 0.80, and 0.90, from which 0.90 was selected for final generalization. All testing statistics in this paper were based on the 390th epoch and the 0.90 detection confidence score, unless noted otherwise.

### 2.4. Whole Core Segmentation and Classification

**Figure 3:**
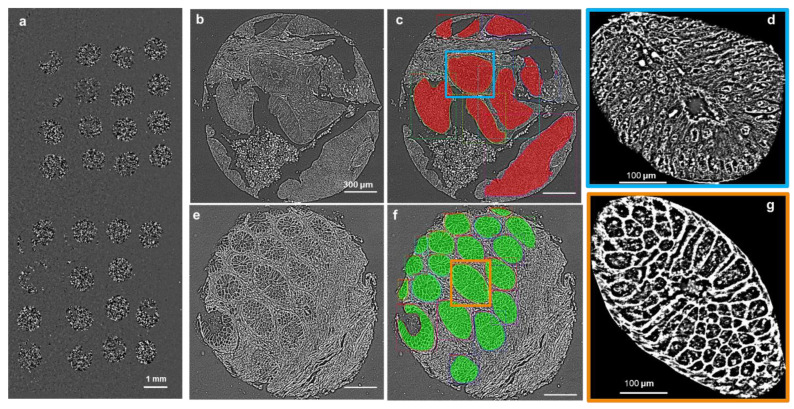
Examples of segmentation and classification. (**a**) Images of the 32 testing cores. (**b**) A cancer core. (**c**) The prediction of gland detection and classification of the core in (**b**). (**d**) A zoomed-in image of the cancer gland boxed in (**c**). (**e**) A normal core. (**f**) The prediction of gland detection and classification. (**g**) A zoomed-in image of the normal gland boxed in (**f**). The red color represents cancer and the green color normal glands.

Since normal glands tend to show clear and regular edges, they can be predicted and classified with higher accuracy. Cancer glands tend to show irregular and less pronounced edges, which makes it more difficult for the network to predict cancer glands. Figure 3 exemplified the results of gland segmentation for both normal and cancer glands. Figure 3b was a cancer core used as an input to the network, while Figure 3c was the output of the network, which displays segmentation, classification, and masking information. Red masks represent cancer glands. Figure 3e,f represent the analog pair for a normal core, where green masks represent normal glands. Figure 3d–g display individual glands from Figure 3c,f, respectively, as indicated.

### 2.5. Overall Gland Classification and Detection

**Figure 4:**
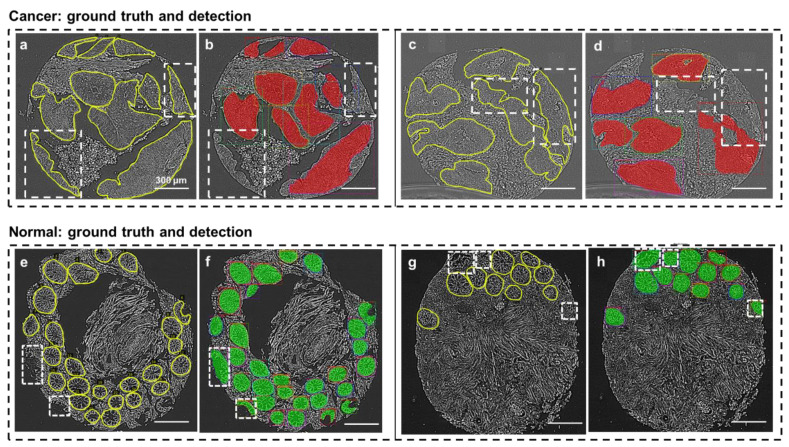
Examples of gland detection errors and additional positive detections. (**a**) Ground truth (manual) segmentation of cancer glands. (**b**) Network predictions showing regions in the dashed boxes that were missed. (**c**,**d**) A similar illustration as in (**a**,**b**). (**e**) Ground truth (manual) segmentation of normal glands. (**f**) Network predictions showing regions in the dashed boxes that were additional true positives. (**g**,**h**) A similar illustration as in (**e**,**f**). Note that all errors and additions occurred at the boundaries of the cell cores.

Figure 4 provides four cases of segmentation output, displaying different performances for *cancer* gland detection and *normal* gland detection. The network segments with higher accuracy for normal glands than cancer glands. In Figure 4a, the network failed to detect two cancer gland areas, which were highlighted by white boxes in Figure 4b. In Figure 4c, two cancer gland areas were also missed, as highlighted by the white boxes in Figure 4d. In Figure 4e,g, all the glands were correctly detected and classified, which can be seen in Figure 4f,h. In addition, two more true normal glands, which were not annotated in the Figure 4e ground truth core and appeared tattered, were detected and classified correctly in Figure 4f; as well as three such cases in Figure 4g,h.

**Figure 5:**
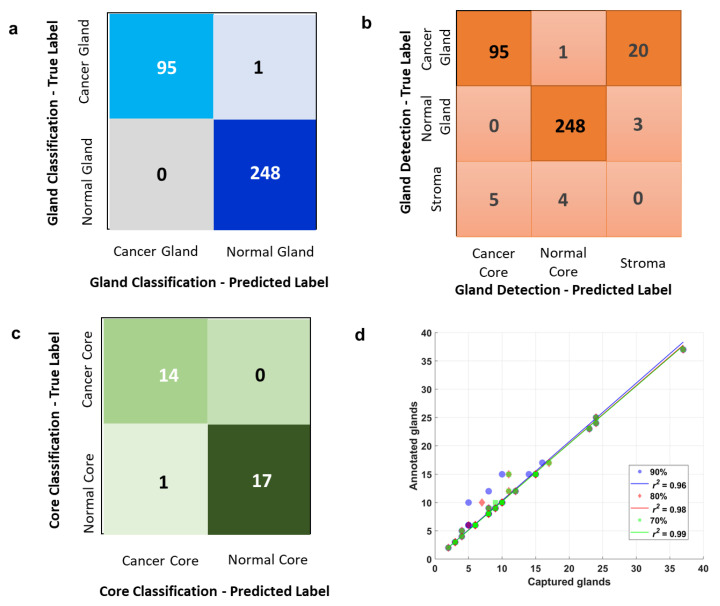
The performance of classification, detection, and diagnosis on a test dataset. (**a**) A confusion matrix which shows that 95 instances of the detected cancer glands were correctly classified, while 1 was wrongly classified as normal. All 248 instances of the detected normal glands were perfectly classified. (**b**) A confusion matrix which shows that 96 cancer glands were detected, with 95 being correctly classified and 1 wrongly classified; 20 cancer glands were missed. A total of 248 normal glands were detected and correctly classified, while 3 normal glands were missed. (**c**) A confusion matrix which shows that all 14 cancer cores were correctly diagnosed as cancer; 17 out of the 18 normal images were correctly diagnosed, while 1 normal core was wrongly diagnosed as cancer. (**d**) Gland detection performance at three different detection confidence scores: 90%, 80%, and 70%, as indicated. The network can capture 96%, 98% and 99% of the annotated glands when its capturing confidence score is set respectively at 90%, 80%, and 99%. Blue dots indicate that the 90% confidence score exerts a highest filtering threshold and thus captures the least amount of glands. The orange dots indicate that the 80% confidence score exerts a lower filtering threshold and thus captures a bit more glands. The green dots indicate that the 70% confidence score exerts the lowest filtering threshold and thus captures the highest amount of glands among the three filters.

Figure 5a,b show the overall performance in gland classification and gland detection by means of a confusion matrix. In Figure 5a, the first row represents the actual class of cancer glands, where 95 instances were correctly classified and 1 instance was wrongly classified as normal. The second row represents the actual class of normal glands, where all of the 248 normal glands that were captured were correctly classified. In Figure 5b, the first row represents the actual class of cancer glands. Of the 116 ground truth cancer glands, 96 cancer glands were detected, of which 1 of the 96 instances was misclassified as a normal gland and the remaining 95 cancer glands were correctly classified. In total, 20 cancer glands were not detected, which were misclassified as stroma or background. The second row represents the actual class of normal glands, of which 248 of the total 251 normal glands were detected and correctly classified, and 3 normal glands were not detected. The third row represents stroma or background, where five stroma tissues were detected and classified as cancer glands, while four stroma tissues were detected and classified as normal glands. Of all the ground truth cancer glands, 83% were detected and 17% were missed. Of all the ground truth normal glands, 99% were detected and 1% were missed. Of all the detected instances, 2% were non-gland instances. Of all the ground truth glands, 6% were not detected.

### 2.6. Overall Core Classification

In our research, each core contained a different number of glands, and a threshold was established for the final classification of cores. If 90%+ of the glands were classified as cancer, this image was diagnosed as cancer. If 90%+ of the glands were classified as normal, this image was diagnosed as normal. The confusion matrix in Figure 5c shows the final diagnosis performance for the test dataset. For ground truth, 14 images were cancer, and 18 were normal. As shown in the first row, all 14 cancer images were correctly diagnosed; as shown in the second row, 17 of the 18 normal images were correctly diagnosed while one was misdiagnosed as cancer.

### 2.7. Detection Performance at Three Different Confidence Scores

Figure 5d shows the network’s performance at three respective gland detection confidence scores, and all of these three scores were generated from the 390th training epoch model. At a 90% detection confidence score, 96% of all the ground truth glands were detected; at an 80% detection confidence score, 98% of all the ground truth glands were detected; and at a 70% detection confidence score, 99% of all the ground truth glands were detected. Of these three scores, the 90% was chosen for the final predictions.

### 2.8. Accuracy Reports in Classification, Detecting and Diagnosis

Table 2, Table 3 and Table 4 are the accuracy reports for detection, classification, and diagnosis, respectively. In the gland detection report (Table 2), the precision score and recall score were 0.95 and 0.82, respectively, in terms of cancer gland detection, and 0.98 and 0.99, respectively, in terms of normal gland detecting. The accuracy in terms of overall gland detection was 0.91%. In the gland classification report (Table 3), the overall classification accuracy for the 344 glands that were detected was 99.71%. The precision score and recall score were 1.00 and 0.99, respectively, in in terms of cancer gland classification. In terms of normal gland classification, the precision score and the recall score were both perfect. In the core classification report (Table 4), the precision and recall scores were 93% and 100%, respectively, in terms of cancer core classification, and 100% and 94%, respectively, in terms of normal core classification; the overall accuracy in terms of whole core classification was 97%.

### 2.9. Detection Performance at Three Different Epochs

**Figure 6:**
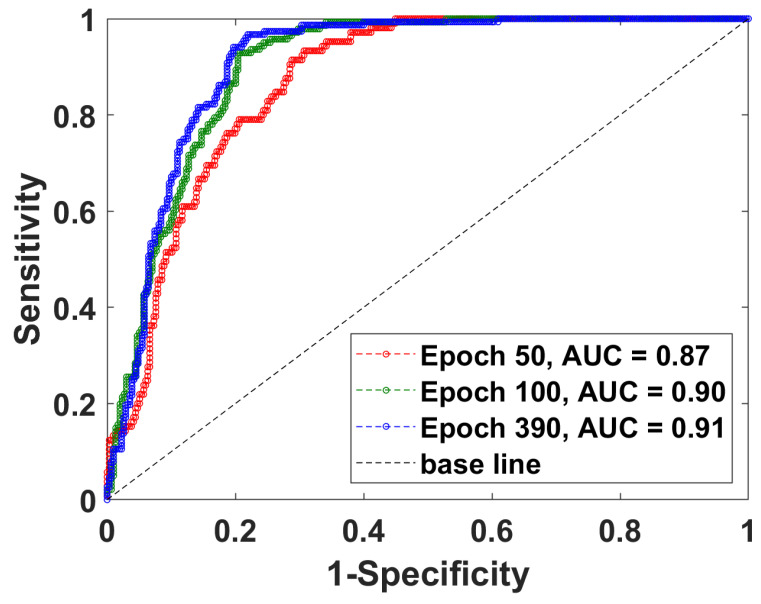
Gland capturing performances at three training epochs. The network’s gland classification performance is shown at three different training epochs, the 50th, 100th, and 390th, as indicated. The AUC (area under the ROC curve) is 0.87, 0.90, and 0.91, respectively.

The network was trained for 500 epochs, of which the first 50 epochs trained only the RPN, classification, and mask heads of the network, while the remaining 450 epochs trained all layers of the network. Three epochs were selected to show that the performance improved slowly from the 50th to the 390th epoch, as illustrated by Figure 6. At the 50th epoch, the network detected 87% of all ground truth glands. At the 100th epoch, the network detected 90% of all ground truth glands. At the 390th epoch, the network detected 91% of all ground truth glands. The improvement pace, though slow, still played a crucial role in core classification, because one error in gland detection or classification might produce an error in core classification.

## 3. Summary and Discussion

In summary, we demonstrated promising results in colorectal tissue segmentation, classification, and whole core diagnosis by combining a mask R-CNN deep learning network with SLIM images. The 91% gland detection accuracy, the near-perfect classification accuracy, and the 97% whole core diagnosis accuracy show that this method can effectively assist pathologists to screen colorectal cancers. Histopathology combined with colonoscopy tissue resection remains the gold-standard for colorectal cancer diagnosis. However, we expect our method to complement valuable pathological information that can improve screening accuracy, reduce manual work, and multiply throughput at clinics. The SLIM module can be integrated with incumbent microscopes across clinics, and then used as a valuable tool to optimize colorectal screening workflows. Moreover, the SLIM module can also be used to quantify the aggressiveness of the cancer disease [16] or detect other types of cancer. For example, molecular markers can be used as non-invasive screening tools in the early stages of colorectal cancer [42]. Once genetic mutations are detected, invasive confirmatory screenings like colonoscopies will follow [42].

With its common-path interferometric geometry, SLIM is highly reliable in providing nanoscale information about tissue architecture, enabling a high sensitivity and specificity for pathological examination. The AI inference method can be integrated with SLIM acquisition software [43]. As the inference runs faster than the acquisition of a SLIM frame and it can also be operated in parallel, we anticipate performing image acquisition and diagnosis in real-time. Theoretically, the diagnosis, with all critical areas of interest being highlighted for pathologists, can be given while the scanning is being done. As part of our future work, we plan to study both dysplastic and hyperplastic tissues, and train these two classes in our deep learning network.

## Figures and Tables

**Table 1:** Training, validation, and testing datasets overview.

	Total Images	Cancer Images	Normal Images	Percentage
Train	196	98	98	76%
Validation	30	15	15	12%
Test	32	14	18	12%
Total	258	127	131	N/A

**Table 2:** The gland detection report on the test dataset.

	Precision	Recall	F1-Score	Support
Cancer Gland	0.95	0.82	0.88	116
Normal Gland	0.98	0.99	0.98	251
Stroma	0.00	0.00	0.00	9
Accuracy			0.91	376
Macro Avg	0.64	0.60	0.62	376
Weighted Avg	0.95	0.91	0.93	376

**Table 3:** The gland classification report on the test dataset.

	Precision	Recall	F1-Score	Support
Cancer Gland	1.00	0.99	0.99	96
Normal Gland	1.00	1.00	1.00	248
Accuracy			1.00	344
Macro Avg	1.00	0.99	1.00	344
Weighted Avg	1.00	01.00	1.00	344

**Table 4:** The core classification report on the test dataset.

	Precision	Recall	F1-Score	Support
Cancer	0.93	1.00	0.97	14
Normal	1.00	0.94	0.97	18
Accuracy			0.97	32
Macro Avg	0.97	0.97	0.97	32
Weighted Avg	0.97	0.97	0.97	32

## Data Availability

The data that supports the findings of this study are available from the corresponding author upon reasonable request.
